# Design and optimization of a metamaterial absorber for enhanced solar cell efficiency and wide band microwave cross polarization conversion

**DOI:** 10.1038/s41598-025-15840-w

**Published:** 2025-08-26

**Authors:** Asmaa S. Mohammed, Ayad Shohdy, Shazly A. Mohammed, Ahmed M. Montaser

**Affiliations:** 1 Faculty of Technology and Education, Sohag University, Sohag, Egypt; 2 Faculty of Engineering, Sohag University, Sohag, Egypt; 3 Faculty of Engineering, South Valley University, Qena, Egypt

**Keywords:** A dual band operation, Metamaterial (MTM) absorption, Harvesting energy, Solar power, Microwave band, Cross polarization conversion (CPC), Electrical and electronic engineering, Devices for energy harvesting, Metamaterials

## Abstract

In this work, the design and construction of a metamaterial (MTM) absorber to increase solar cell efficiency is proposed. MTM is use as frequency selective surface (FSS) in the infrared band. The design is made up of a split ring resonator (SRR) imprinted on the substrate’s top surface, with a copper layer serving as a ground on the back layer of the substrate material. The structure works at tera frequencies to take in all the sun’s infrared wavelengths. Furthermore, a MTM array absorption is formed by using the newly generated MTM unit cell, which improves harvesting energy from the sun spectrum. Both designs demonstrated absorption rates of roughly 99% at resonance frequency 13.29 THz. The parameters of the proposed unit cell are also edited and optimized to operate in the microwave frequency ranges where the wide-band microwave cross polarization conversion (CPC) metasurface (MTS) is simulated, fabricated and validated. The measured data much agrees with the simulation one. The suggested CPC MTS achieves efficient cross-conversion over a large frequency range (19.6–25.9 GHz), with a polarization efficiency of 90% and two bands operation. It has a fractional bandwidth (FBW) of 27.7%. The polarization interaction is stable up to 45° oblique incident angle.

## Introduction

Based to the 2014 Global Energy Statistics Report, fossil-fueled (traditional) energy sources such as petroleum and coal account for 87% of total global energy consumption^1^. This large percentage has a negative impact on ecosystems and makes fossil fuel production difficult. Finding a replacement for clean, consistent, satisfying, and recyclable energy sources is so critical. The sunlight is one of the greatest efficient sources of clean and renewable energy, creating light, heat, lighting, and other forms of energy^2–4^. Solar cells, commonly referred to as solar panels, produce power using sunlight. Solar cells are commonly made of silicon, a substance that comes into contact with photons of sunlight inside the cell, causing atoms to lose their electrons from the semiconductors and form electrical gaps that produce energy^5,6^. The fundamental advantage of any photovoltaic panel-based structure is its capacity to run without the need for battery replacement on occasion. Nonetheless, these cells are capable of absorbing certain frequency bands of solar light, yielding 30% efficiency. The electromagnetic (EM) range gap, which prevents cells from converting all solar light into power, is the cause of this low efficiency^7^.

Microwave absorbing materials with the properties of thinness, lightweight, broadband, and strong absorption have received rising attention because of their pragmatic and effective functions for reducing electromagnetic interference (EMI) pollution and defense stealth technology. Recently, metamaterials (MTM) and metasurfaces (MTS) technologies have been used. This technology offers many advantages such as smaller size, wider bandwidth, and better radiation properties. Moreover, it suppresses surface wave excitation that can degrade the radiation properties of the antenna^8^. MTM is made with unique properties not found in nature. Because of their intelligent qualities, MTM is organized in periodic patterns on scales smaller than the spectral range of the processes they influence. It can process EM waves^9^, blocking, bending, or absorbing waves^10–12^ and change the elastic properties of materials. Furthermore, applications span numerous areas, including public safety, high-frequency combat communications, remote aerospace applications, sensor identification, and military research for detecting explosives and pollution^8,13^. Additionally, it has negative refract characteristics^14^, allowing it to function as ultra-lenses^15^. According to the literature, MTM absorbers have been the focus of many studies and searches by various experts. MTM absorbers can be displayed in a variety of ways^16–18^. In^19^ a novel scheme was proposed to realize a sub-terahertz meta-device with triple-band filtering using a conductive coupling strategy to excite a strong interaction between two identical sub-resonators. Three filter slopes located at 0.238 THz, 0.504 THz, and 0.636 THz were experimentally obtained, which are consistent with the theoretical results, and the near-field and surface current enhancement of these filter slopes were extracted to explore their mechanisms. In^20^ a simple design of a meta-terahertz metamaterial that can produce a three-band EIT effect using the coupling of the “bright big” modes of two sub-resonators is proposed. Three transparency peaks around 0.59 THz, 1.07 THz, and 1.34 THz are experimentally realized, and their formation mechanisms are explored. The design of MTM-based absorber described in^21^ incorporates an infinite number of multi-resonance particles on the nanoscale. The absorber may absorb a substantial amount of solar light. The concept of collecting solar energy from sun and earth radiation using metamaterial absorber is based on the fact that when an EM wave is incident to an absorber, a time varying current will be induced, and thus, a voltage will be generated at the feeding point of the absorber^22,23^. This induced current or generated voltage will oscillate at the frequency of the incident wave. Hence, in order to obtain DC power, a suitable rectifier circuit should be embedded at the feed point of absorber. This type of energy harvesting systems are called rectennas and consist of antennas connected to a rectifier that converts the received signal to DC power and produce electricity. The metamaterial absorber shows a wider angular reception characteristic than that of photovoltaic devices. This optimizes the solar energy collection during day and cancels the need for sun tracking systems^24^.

MTM-based solar cells are intended to absorb the visible and infrared spectrums optimally. Furthermore, several novel and unorthodox strategies for increasing antenna arrays and MTM properties in the vast majority of antennas and materials have been proposed^25–28^. One of these strategies is utilized to improve the performance of the suggested MTM antenna arrays by executing the value scan on the most essential and effective values that define the MTM antenna array’s properties, such as rate of absorption. In addition, a structure consisting of super-absorbing material is built for harvesting energy from the sun, with rates of absorption nearing 99%^29^.

Many fascinating phenomena are inherently sensitive to polarization states. These have historically been one of the most important features of EM waves, and significant attempts have been made to control and manage them^30–33^. MTM, especially plane MTM, has recently received a lot of attention because of their unique properties. Planar optics^34^ has a wide range of applications, including anomalous refraction ^35^, angular momentum of light^36^, optical vortex formation^37^, polarization conversion (PC)^38^, and transmission and reflection types^35–41^. Because several phenomena are polarized sensitive, a large portion of MTS research focuses on surfaces that can regulate and change the polarization of EM waves. While traditional methods such as optically crystal activity and the Faraday effect can be used to change the polarization of EM radiation, they require a large volume and are only effective over a small bandwidth^42^. As a result, EM polarization states can be flexibly controlled and manipulated by using sub-wavelength MTM. Two-dimensional MTM, also known as MTS, are utilized to build powerful polarization converters^33^. PC has been accomplished over several frequencies bands bsystem^43^, circular divided bands in the infrared range^44^, metallic grating at terahertz^35^, and self-complementary bands^45^, a rectangle cycles with opposite microstrips^46^, and double-head-arrow form^47^ in the microwave frequency system^48^.

This study proposes and presents a unique absorber design based on MTM technology for improving solar cell efficiency. A minimal structural unit cell composed of two separate sheets with rectangle gaps of constant size can absorb a significant amount of infrared radiation at different tera hertz. Additionally, this unit cell is used for creating arrays of various dimensions with the identical infrared frequencies of resonance in order to absorption as many photons as possible in the spectrum of the sun. The array is also suitable for microwave applications and the proposed design is edited to operate as a microwave CPC MTS and it can give a wide bandwidth via plasmon resonance frequency. The suggested microwave CPC MTS unit cell structure is based on a synthetic absorber design identical to a solar cell, but in the microwave range, and the CPC is useful under normal and atypical conditions. The suggested wide-band and electrically compact design is also polarized at microwave frequencies, where the polarization efficiency of conversion is great and completely independent of the incident wave’s polarization. In addition, the proposed upper surface provides a steady reaction to variations in the incidence angle. Because of its sub-wavelength unit cell size and thin dielectric thickness, considering overall ideal design, the CPC MTS contact is independent of the incoming wave’s incidence angle, making it a viable competitor for a variety of applications.

## Design and analysis

### Design of the proposed MTM unit cell in the infrared band

**Fig. 1:**
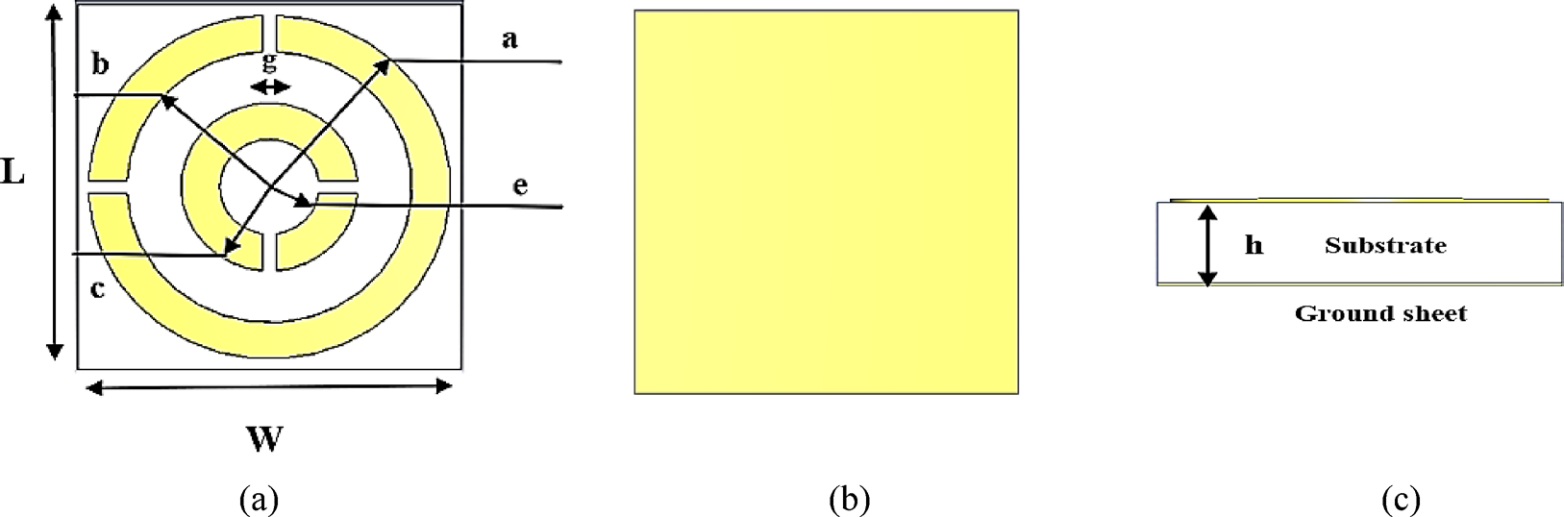
Proposed designed absorber unit cell (**a**) 2D- front view, (**b**) 2D- back view, (**c**) side view.

The proposed unit cell architecture is based on prior research and analysis presented in literature^3–10,42^ affected both the reference shape and the overall concept. The reference’s shape has been modified to make it simpler, more compact, and capable of dual band operating. Figure 1 depicts a diagram of the proposed unit cell. The unit cell is made of two copper sheets divided by a 0.76 mm thick Rogers 4350B substrate (dielectric constant *ε*_r_ = 3.66, loss tangent tanδ = 0.0037). The top copper layer is a split ring resonator (SRR) which composed of printed circular slices with rectangular gaps of 0.1 µm on the substrate’s top surface. The back layer is supported by a copper ground plate with about 0% transmission, measuring 3 × 3 µm in infrared. The copper layers have a conductivity of 5.8 × 10^7^ S/m and a thickness of 0.0035 µm. The suggested unit cell undergoes thorough parameter checks to ensure that it performs properly in the defined band frequencies. The optimal parameters are given in Table 1. This approach combines two separate components to form a unit cell. A single of the two separate pieces has a negative comparative permeability, whereas the other has a negative comparative permittivity. This method is predicated on the assumption that resonance-sensitive contents show extreme values for effective permeability to magnetic fields near the resonance when exposed to an axial magnet, with substantially positive results in the narrow range beneath the rings’ quasi-static resonance frequency and highly negative results in the narrow range above^49^.

**Table 1:** The geometrical dimensions of the proposed infrared absorber and CPC microwave unit cell.

Description	CPC MTS	Infrared unit cell	Parameter’s list
	Value (mm)	Value (µm)	
The substrate material’s width	5	3	W
The substrate material’s length	5	3	L
Thickness of the substrate material	0.76	0.76	h
Rectangular gap width	0.125	0.1	g
The outer radius of the outer slice	2	1.41	a
The inner radius of the outer slice	1.5	1.11	b
The outer radius of the entire slice	1	0.68	c
The inner radius of the entire slice	0.5	0.38	e

### The theory

The absorption rate *A*($\upomega$) of solar cells can be calculated using^5^:

1 $$A\left( {\upomega } \right) = 1 - R\left( {\upomega } \right) - T\left( {\upomega } \right)$$

The reflectivity and transmission at a certain frequency range are represented by ($\upomega$) =|*S*_11_|^2^ and *T*($\upomega$) =|*S*_21_|^2^, respectively. The continuous copper sheet prevents the calculation of a single transmission (*T*($\upomega$) → 0) in this study. As a result, just the reflection should be analyzed. As a result, the absorption rate is modified and computed as follows:

2 $$A({\upomega })\, = \,{1}\, - \,R({\upomega })$$

The reflectance can be expressed as follows:

3 $$R({\upomega }) = \frac{{{\text{Reflected power at port }}1}}{{{\text{Incident power on port }}1}}$$

As reflected decreases, the rate of absorption increases and approaches unity, which happens to be the main purpose of this study. This is proven with the use of an MTM absorber.

### Geometric configuration of the CPC microwave unit cell

#### Unit-cell structure

**Fig. 2:**
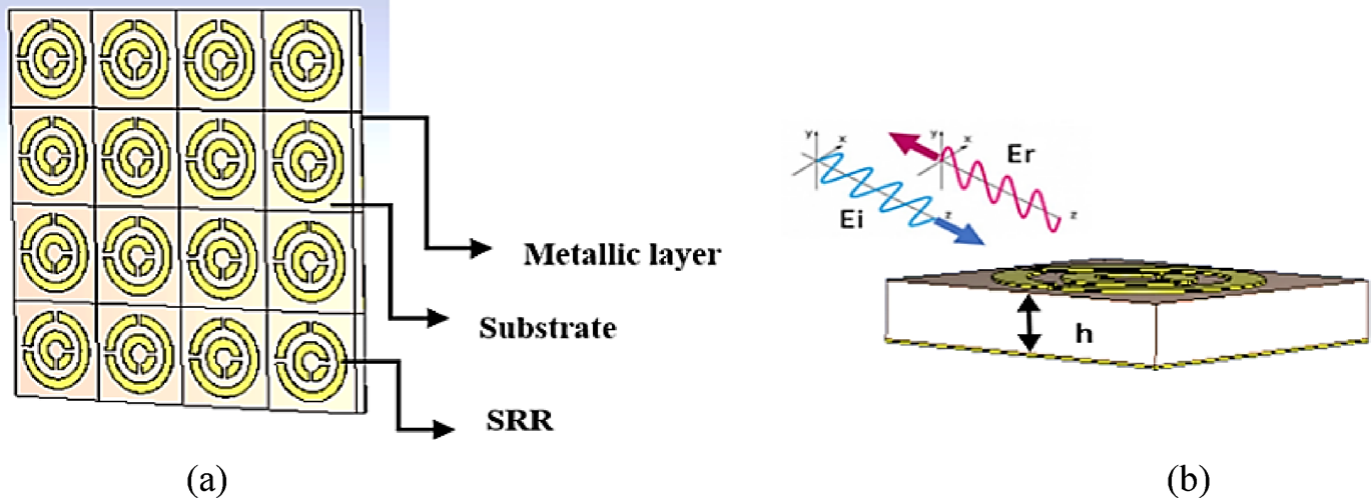
(**a**) A general schematic depiction of the proposed CPC MTS. (**b**) 3D- view.

The design parameters of the proposed infrared unit cell are edited and optimized to operate in the microwave frequency ranges where a wide-band microwave CPC MTS is conceived, simulated, fabricated and validated. The optimal parameters of the designed unit cell are also given in Table 1 for comparison. Figure 2a shows the two-dimensional regular array of metallic resonances, with split ring resonators (SRRs) mounted on the top of the dielectric and held up by a metallic plane. Figure 2b displays a three-dimensional schematic representation of the unit cell.

#### Analysis based on Jones reflection matrix

Jones transmission and reflection matrices help to define the theory of PC. Transmission is absent in a reflector design, therefore only incident and reflected fields are considered. The wave polarization is described in terms of its orthogonal components, and the degree of PC is determined by comparing the co- and cross-components of the entering and reflected fields. The anisotropic structure of the unit cell interactions differently with the two perpendicular elements, causing cross-conversion. Consider an incoming wave that is vertically polarized and travels along the positive *z* axis before striking a MTS reflection at its normal incidence. Equation (4) denotes the wave’s EM field vector ^50–52^.

4 $$\overrightarrow {{E^{i} }} = \left| {\overrightarrow {{E_{x}^{i} }} } \right|e^{{ - j\varphi_{xi} }} { }\vec{e}_{y}$$

The superscript *i* represents the occurrence field, whereas the subscript y indicates the vector’s constituent label. ${\varphi }_{i}$ is the direction angle of the field’s phasor. ${\overrightarrow{e}}_{y}$ denotes the unit vectors in the *y* axis. The electrical field vectors might spin in the *x–y* plane after reflection from the interaction, therefore both orthogonal components, as given in Eq. (5a), are required for an exhaustive account of the reflected wave. Equation (5b) presents these component energies as complex reflection coefficients.

5a $$\overrightarrow {{E^{r} }} = \left| {\overrightarrow {{E_{x}^{r} }} } \right|e^{{ - j\varphi_{x} }} { }\vec{e}_{x} + \left| {\overrightarrow {{E_{y}^{r} }} } \right|e^{{ - j\varphi_{x} }} { }\vec{e}_{y}$$

5b $$\overrightarrow {{E^{r} }} = \left| {\overrightarrow {{E_{y}^{i} }} } \right|\left( {R_{xy} e^{{ - j\varphi_{x} }} { }\vec{e}_{x} + R_{yy} e^{{ - j\varphi_{y} }} { }\vec{e}_{y} } \right)$$

In the preceding equations, a superscript *r* denotes the mirrored field, while subscripts *x* and *y* represent field constituent labels. The Jones reflections matrix, depicted in Eq. (6), displays the overall link among the polarized linear incident and reflective fields.

6 $$\left[ {\begin{array}{*{20}c} {E_{xr} } \\ {E_{yr} } \\ \end{array} } \right] = \left[ {\begin{array}{*{20}c} {R_{xx} } & {R_{xy} } \\ {R_{yx} } & {R_{yy} } \\ \end{array} } \right]\left[ {\begin{array}{*{20}c} {E_{xi} } \\ {E_{yi} } \\ \end{array} } \right] = R_{lin} \left[ {\begin{array}{*{20}c} {E_{xi} } \\ {E_{yi} } \\ \end{array} } \right]$$

${R}_{lin}$ commonly known as the Jones Reflection Matrix, defines the wave reflection phenomenon in polarized linear waves. The co-polarization coefficients of reflection ${R}_{xx}$ and ${R}_{yy}$ are expressed as the ratio of the amounts of horizontally reflected with horizontally incidence fields $({R}_{xx}=\left|{E}_{xr}\right|/\left|{E}_{xi}\right|)$ and vertically reflected to vertical incidence $({R}_{yy}=\left|{E}_{yr}\right|/\left|{E}_{yi}\right|).$ Similarly, ${R}_{xy}$ and ${R}_{yx}$ are cross-polarization coefficient that connect the cross-field components, i.e. $({R}_{xy}=\left|{E}_{xr}\right|/\left|{E}_{yi}\right|)$ and $({R}_{yx}=\left|{E}_{yr}\right|/\left|{E}_{xi}\right|)$.

#### Design development

**Fig. 3:**
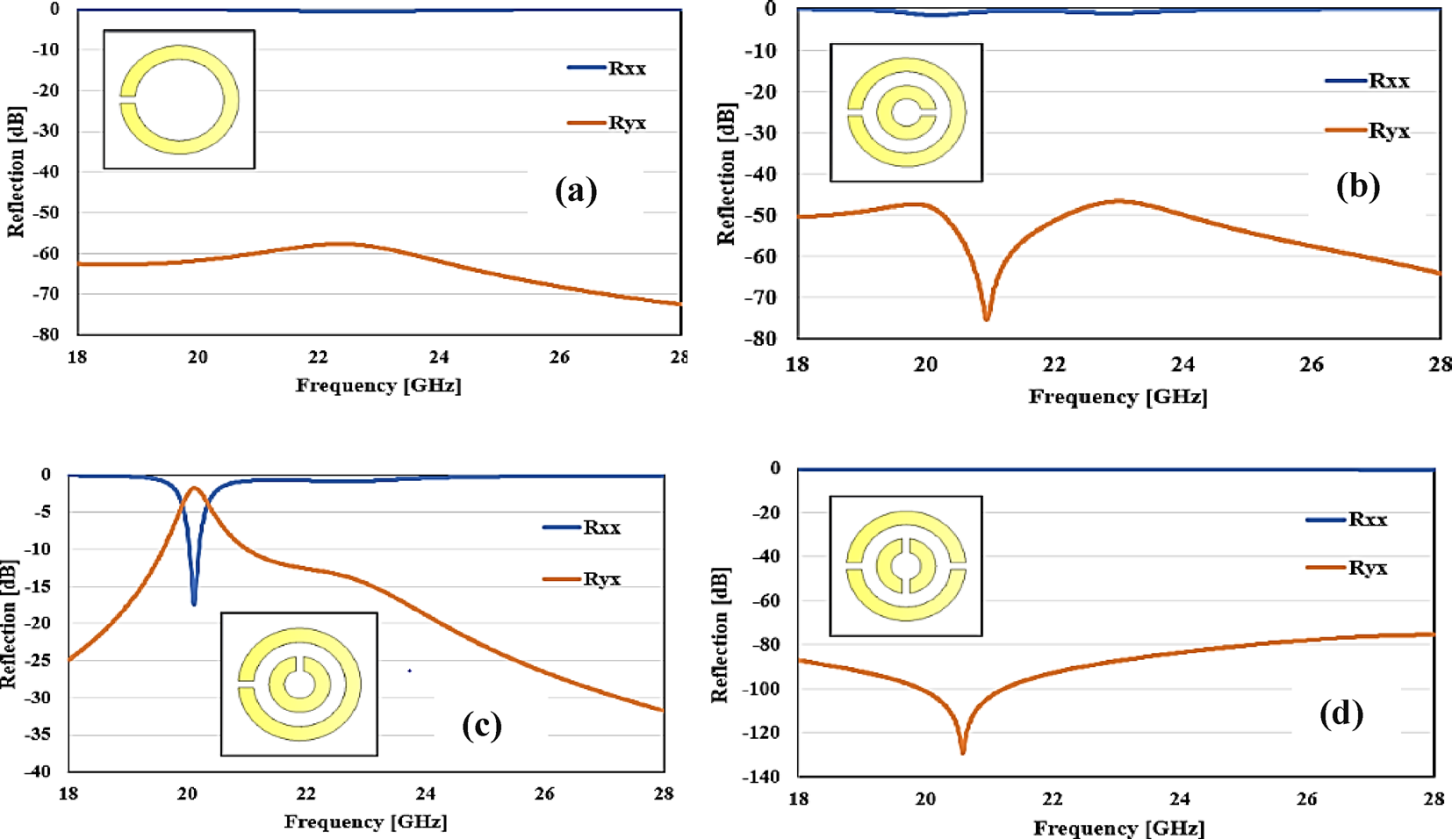
Co- and cross-polarization reflection coefficients for (**a**) Single SRRs and single splits, (**b**) Single split linked SRRs with splits located opposite one another, (**c**) Single split linked SRRs with splits that are perpendicular to one another, and (**d**) Coupled SRRs each have two splits. The splits in each SRR are arranged opposite one other, all the parameters are identical to those for the optimized design in Fig. 2b.

Figure 3 depicts the simulation results of the intermediary design stage, which can be used to comprehend the design method. While the previous intermediary creates, particularly those depicted in Fig. 3b–d, are quite similar to our ultimate optimized design (Fig. 2), neither of them achieve complete CPC. The SRR-based design illustrated in Fig. 3a produces just a common polarization refraction, in which the incoming *y*-polarized field (as well as the split loaded edge) induces currents of electricity not just in the sides through the *y*-axis additionally through the *x*-axis (no divided loaded edges); nevertheless, the electric currents at the positions along the *x*-axis are in opposition of each other, canceling each other out, and no cross-polarization reflection is achieved. If the reflected fields are *x*-polarized (no divided loaded edges), the current will only circulate along the *x*-axis, leaving no cross-polarization reflections. Figure 3b, c show unit cells with two SRRs, although CPC is still impossible since single SRRs have only one split. Even though a unit cell shown in Fig. 3d includes two divides for every SRR, because they are on opposite ends, they cannot generate CPC because an* x*-polarized field cannot cause net current to move in the plane of *y*, and a *y*-polarized field cannot cause net current to move in the *x* direction. To facilitate cross-polarization converting, each SRR requires two divides, which must be positioned on the opposite edges of the SRR, as shown in our ideal design.

This proposed structure has one split-bearing and one non-split-bearing side for each axis. As a result, an incident *x* or *y*-polarized field continually comes into contact with a split-bearing side, causing current to move not just on that side but also on the opposite no split-bearing side. Because the electric currents in the divided and non-split-bearing sides diverge in phase, during reverberation, the net current flowing in the opposite direction of the applied electric field is greatest, leading in a successful CPC.

## Results and discussion

### Results and discussion of the proposed MTM unit cell in the infrared band

**Fig. 4:**
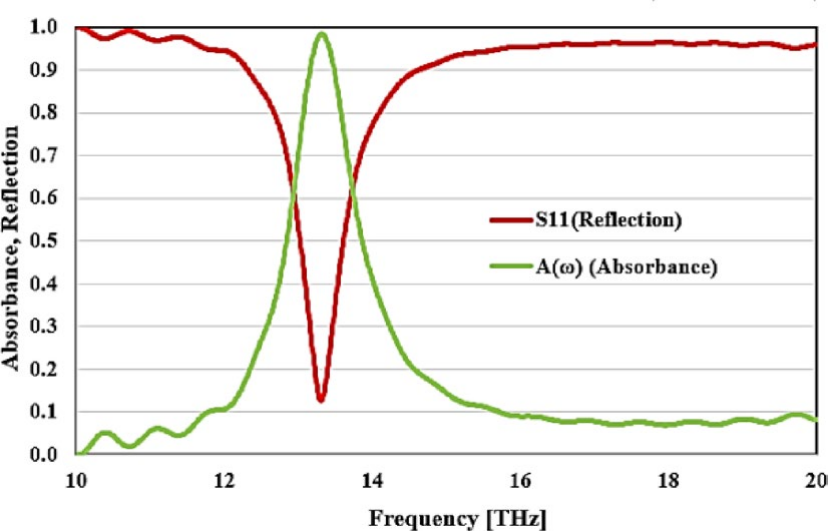
Simulated reflection and absorption rate of the designed absorber unit cell at the infrared radiation.

The proposed structure is simulated using the computer simulation technology microwave studio simulator (CST-MWS). The results of the simulation are shown in the infrared range, with a frequency band of 13.15–13.48 THz. The proposed structure has a single absorption peak at the principal resonance frequency. The results show that a highest absorption peak of 99% occurs at the resonance frequency of 13.29 THz as shown in Fig. 4 where *A*(ω) is the absorption rate, while S11 is the reflection parameter.

**Fig. 5:**
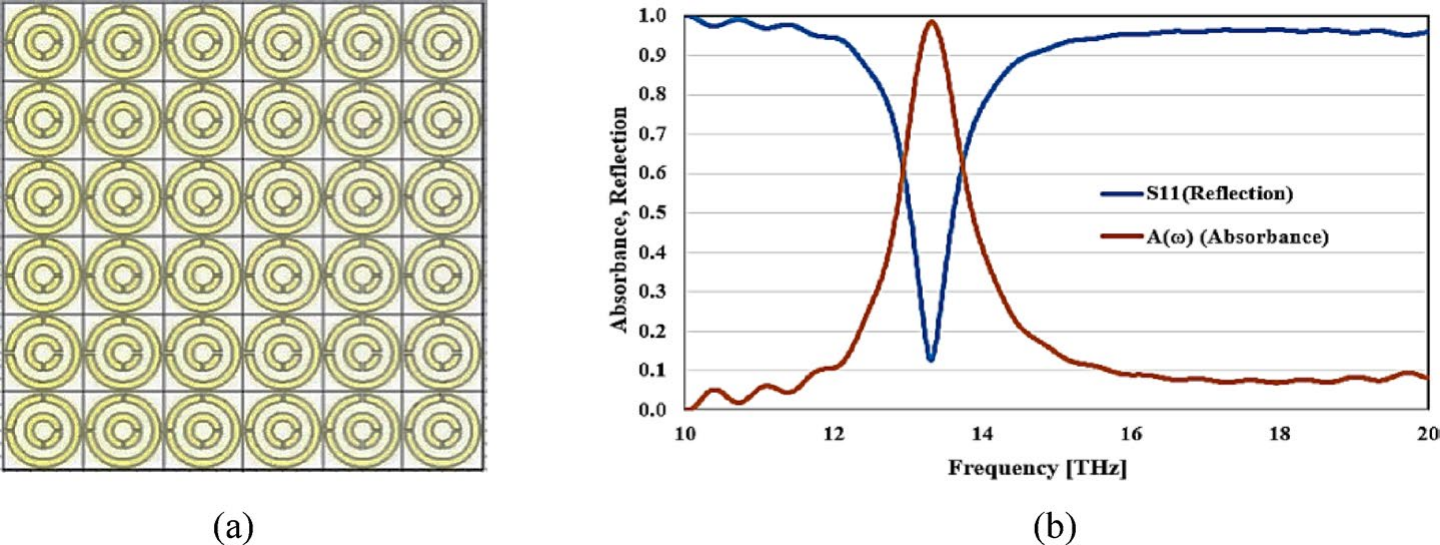
(**a**) Designing and constructing a unit cell array, (**b**) Simulated reflection and absorption rate of unit cell array.

Figure 5 displays the structure and construction of a unit cell array intended to collect and absorb as much solar infrared energy as feasible. It also shows the simulation results of this matrix’s absorption and reflection coefficients. Based on the information provided, it is clear that the absorption rate and excellent performance of the unit cell remain consistent.

**Fig. 6:**
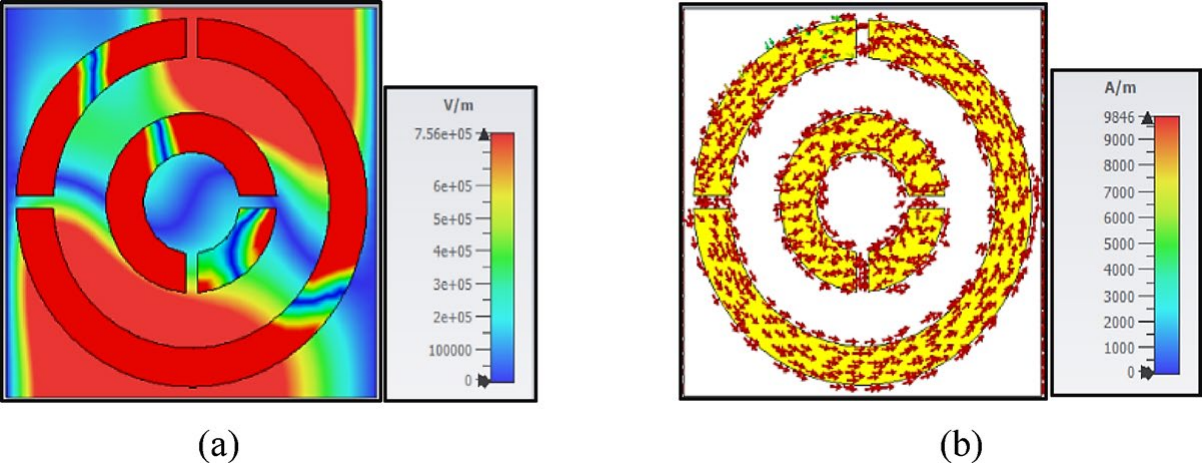
(**a**) The distribution of the electric field in the unit cell at 13.29THz, (**b**) The distribution of the surface current in the unit cell at 13.29THz.

The Fig. 6 illustrates the distribution of the electric field and surface current within a unit cell at a frequency of 13.29 THz. Here’s a detailed explanation of the distributions:

#### Distribution of the electric field

*Electric field distribution* The electric field distribution within the unit cell at 13.29 THz is depicted in part (a) of the figure. The electric field lines or contours show how the electric field varies spatially within the unit cell.

*High field regions* Areas with higher electric field intensity are typically indicated by brighter or more densely packed contours. These regions often correspond to areas where the electromagnetic energy is concentrated, such as near sharp edges, gaps, or resonant structures within the unit cell.

*Low field regions* Areas with lower electric field intensity are shown with darker or less dense contours. These regions indicate where the electric field is weaker, often in areas further from the resonant structures or in regions where the field is more uniformly distributed.

*Field direction* The direction of the electric field vectors would indicate the orientation of the electric field at different points within the unit cell. This can provide insights into how the field interacts with the structure and how it propagates through the unit cell.

#### Distribution of the surface current

Surface Current Distribution: Part (b) of the figure shows the distribution of surface current within the unit cell at the same frequency (13.29 THz).

High Current Regions: Areas with higher surface current density are typically indicated by brighter or more intense colors. These regions often correspond to the paths where electrons are more freely moving, such as along conductive surfaces or at the edges of metallic structures.

Low Current Regions: Areas with lower surface current density are shown with darker or less intense colors. These regions indicate where the current flow is minimal, often in areas where the conductive paths are less direct or where the current is more spread out.

Current Direction: The direction of the current flow shows how the current moves across the surface of the unit cell. This can help in understanding the flow of charge and the formation of current loops or other patterns that are critical for the electromagnetic response of the structure.

The electric field distribution (a) highlights how the electromagnetic energy is concentrated and distributed within the unit cell, with high-intensity regions indicating areas of strong field interaction. The surface current distribution (b) shows how the current flows on the surface of the unit cell, with high-density regions indicating paths of significant electron movement. These distributions are crucial for understanding the electromagnetic behavior of the unit cell at the specified frequency, which is essential for designing and optimizing metamaterials or other periodic structures for specific applications.

### Results and discussion of the CPC microwave unit cell

#### Results from simulations and parametric analysis

**Fig. 7:**
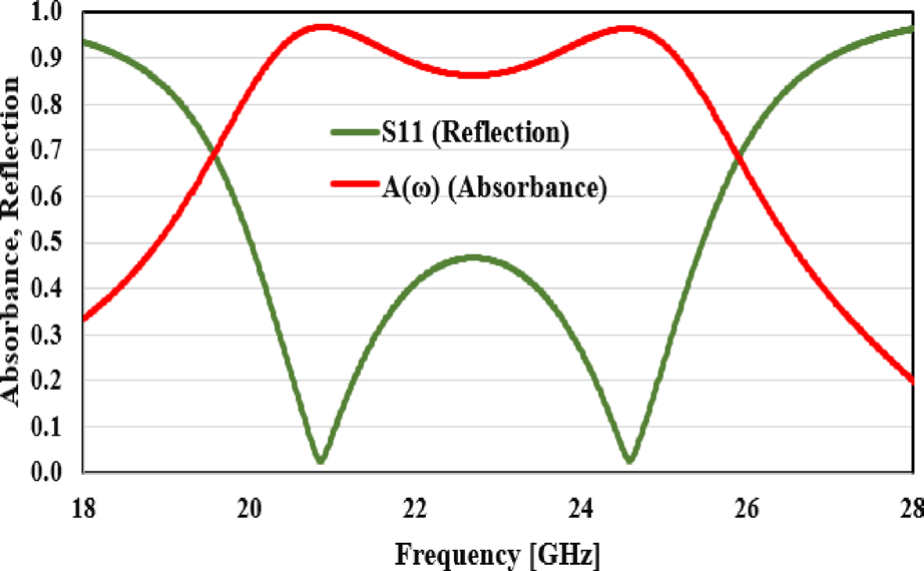
The absorbance and reflection results in microwave range.

**Fig. 8:**
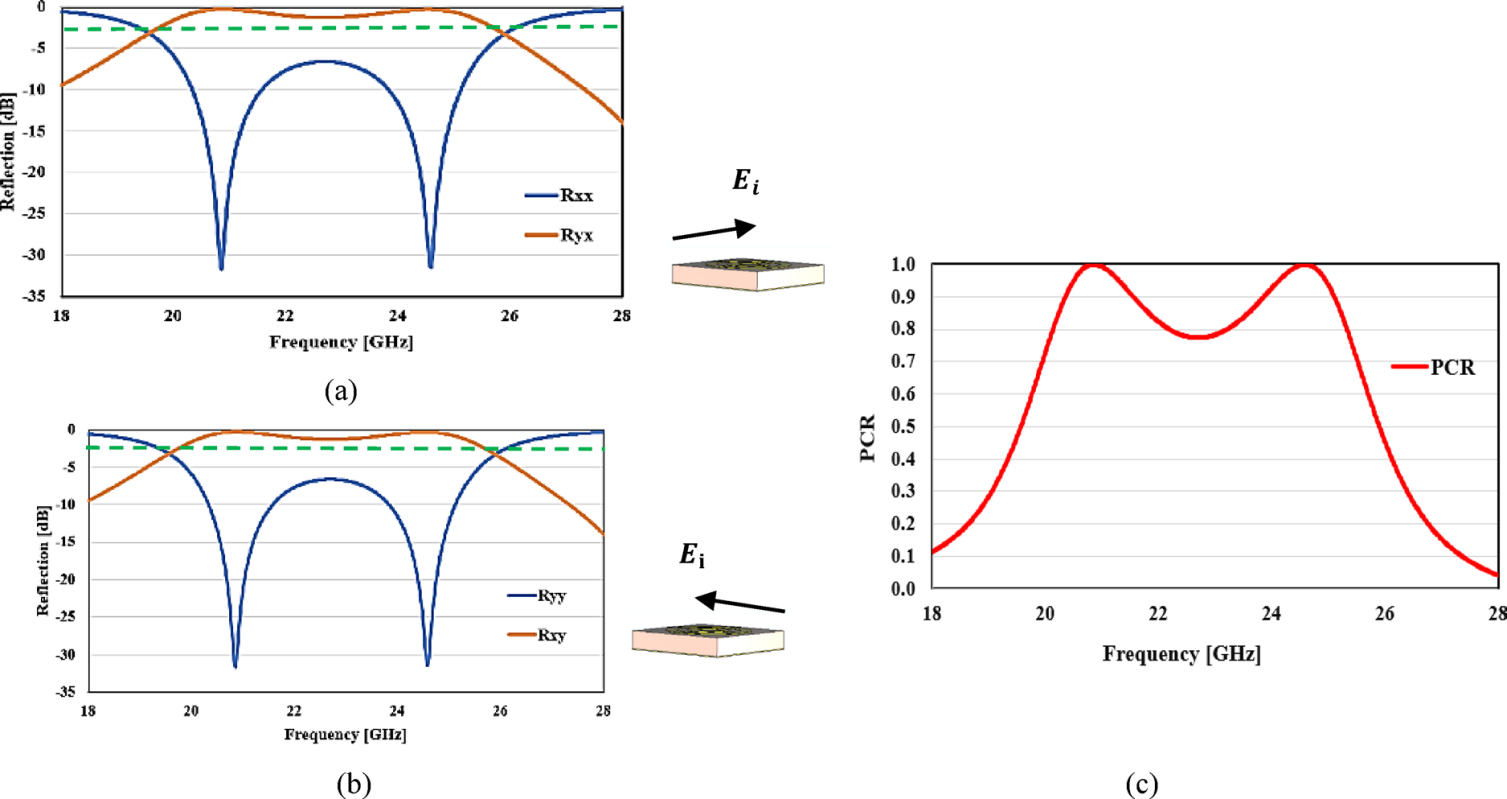
The values of cross- and co-polarized reflection coefficients for (**a**) x-polarized, (**b**) y-polarized incident waves and (**c**) PCR.

The simplified design depicted in Figs. 7 and 8 is handled by full-wave simulations utilizing the CST-MWS simulator. To validate the MTM results, a MTS in the microwave region is created. Figure 7 depicts the microwave-based reflection and absorbance results. Figure 8a depicts simulated co-and cross-polarized coefficients of reflection for typical incidence with an *x*-polarized incident field. Figure 8a shows that the co-polarized reflection coefficient is negligible, whereas the cross-polarized reflection value is more than − 3 dB in the (19.6–25.9 GHz) frequency region. A 27.7% fractional and-width allows for a broad 3 dB bandwidth at resonant frequencies of 20.84 GHz and 24.58 GHz (19.6–25.9 GHz). As illustrated in Fig. 8a, resonant frequencies occur at 20.84 GHz and 24.58 GHz respectively. At all of these resonances, cross polarization is at its highest and co-polarized reflection coefficient is minimized.

Figure 8b depicts co-and cross-polarized coefficients of reflection for typical incidence when the incident field is *y*-polarized. The co-polarized reflection coefficient is minimal, similar to that of *x*-polarized wave; however, the cross-polarized coefficient of reflection is greater than − 3 dB in the frequency band of (19.6–25.9 GHz). A *y*-polarized wave also has a broad 3 dB bandwidth at resonant frequencies of 20.84 GHz and 24.58 GHz (19.6–25.9 GHz). Figure 8a,b illustrate that the MTS responds similarly to both *x* and *y*-polarized waves; thus, these polarizations are transformed into cross-polarized waves in a comparable manner. The symmetry of the unit cell in the *xy* plane contributes to the MTS’s polarization insensitivity. To determine the efficacy of PC, we utilize a statistic known as the polarization conversion ratio (PCR), which is defined as PCR =|${R}_{xy}$|^2^/(|${R}_{xy}$|^2^ +|${R}_{yy}$|^2^). It is the power of the reflected polarization element divided by the total reflected power. For flawless cross-conversion, use PCR = 1 or conversion efficiency = 100%. Figure 8c shows the PCR of the proposed top surface assuming its natural occurrence. At the resonance frequencies of 20.84 GHz, and 24.58 GHz, almost perfect PCR of more than 90% are achieved, and the reverse is true.

#### Angular stability

**Fig. 9:**
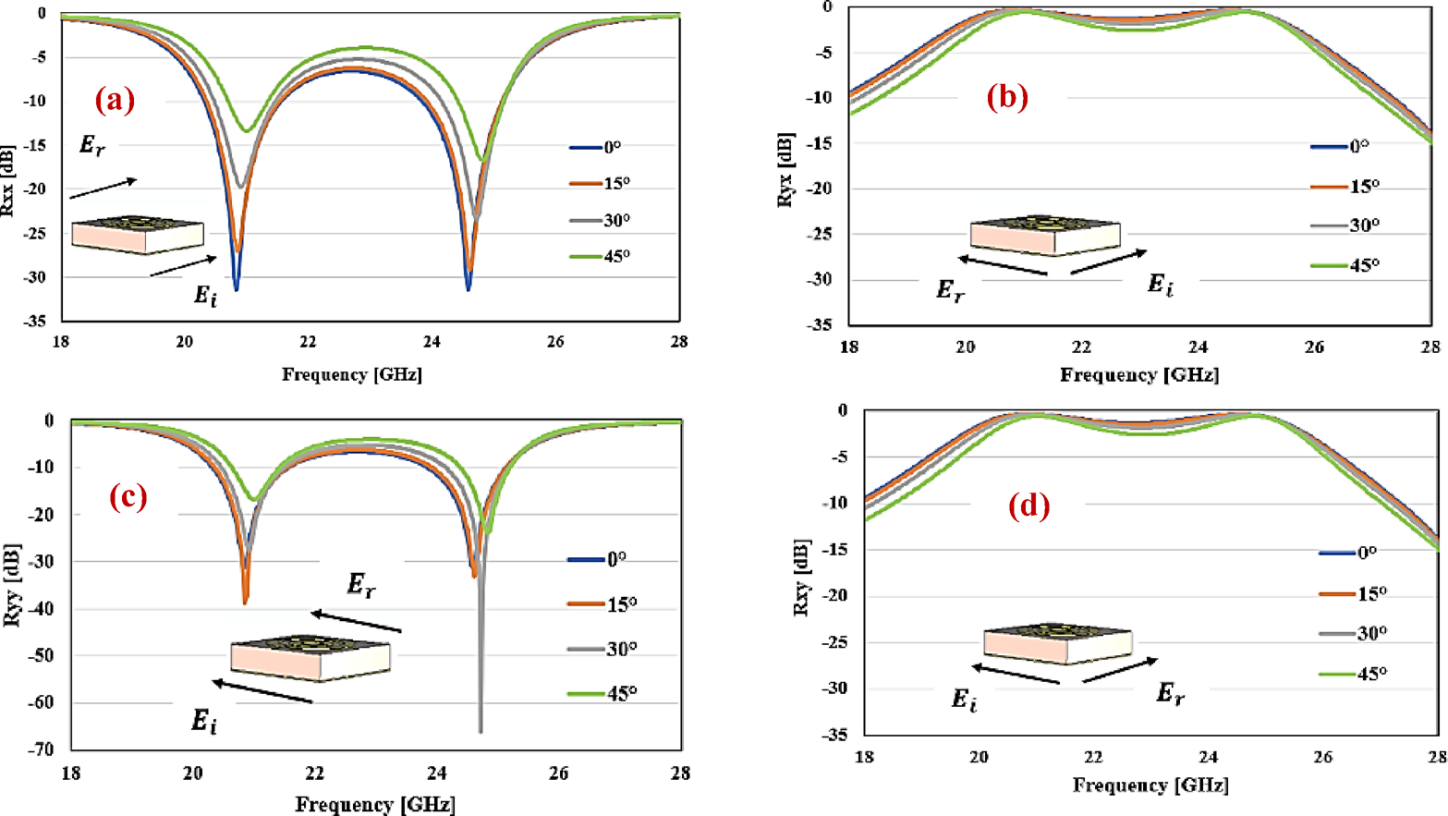
(**a**) Co-polarized and (**b**) cross-polarized reflection coefficients for *x*-polarized incident wave, (**c**) Co-polarized and (**d**) cross-polarized reflection coefficient for the *y*-polarized incident wave.

The CPC MTS to be useful in a wide range of applications, the MTS’s reaction must be consistent enough to withstand fluctuations in angles of incident. The angular stability of the MTS is often proportional to the substrate’s thickness and the dielectric constant. MTS with thin insulating substrate exhibit improved angular resilience. The smaller the MTS in relation to the incident wavelength, the more robust its reactivity to changes in angles of incident; however, the band width decreases. Another way to achieve stability of angle is to employ a dielectric with a high dielectric constant, which decreases bandwidth. Angular stability, bandwidth, and MTS thickness are typically traded-off. A potential technique for achieving wide band and stability of angle is to carefully construct a metal frame on the highest of the insulator so that the unit cell can experience many resonances. Figure 9 shows the co- and cross-polarized coefficients of reflection at different incidence angles.

Figure 9a, b demonstrate the co- and cross-polarized coefficient of reflection at various incident angles when the incident waveform is *x*-polarized. The cross-polarized reflection coefficient, shown in Fig. 9b, remains nearly constant as the angle of incidence varies. The modest divergence in the cross-polarized coefficient of reflection for the *x*-polarized condition at a broad angle of incidence is caused by an electric field component entering the SRR loop and influencing current flow. Figure 9c, d depict the co- and cross-polarized coefficient of reflected when the wave that is reflected is *y*-polarized at varying incident angles. The proposed design, illustrated in Fig. 9d, produces a consistent cross-polarized coefficient of reflection even when the incident waves are *y*-polarized. The slight change in the cross-polarized coefficient of reflection for the *y*-polarized condition at high incident angles is due to a field of magnets that enters the SRR cycle and induces current in it.

#### Analysis based on surface current

**Fig. 10:**
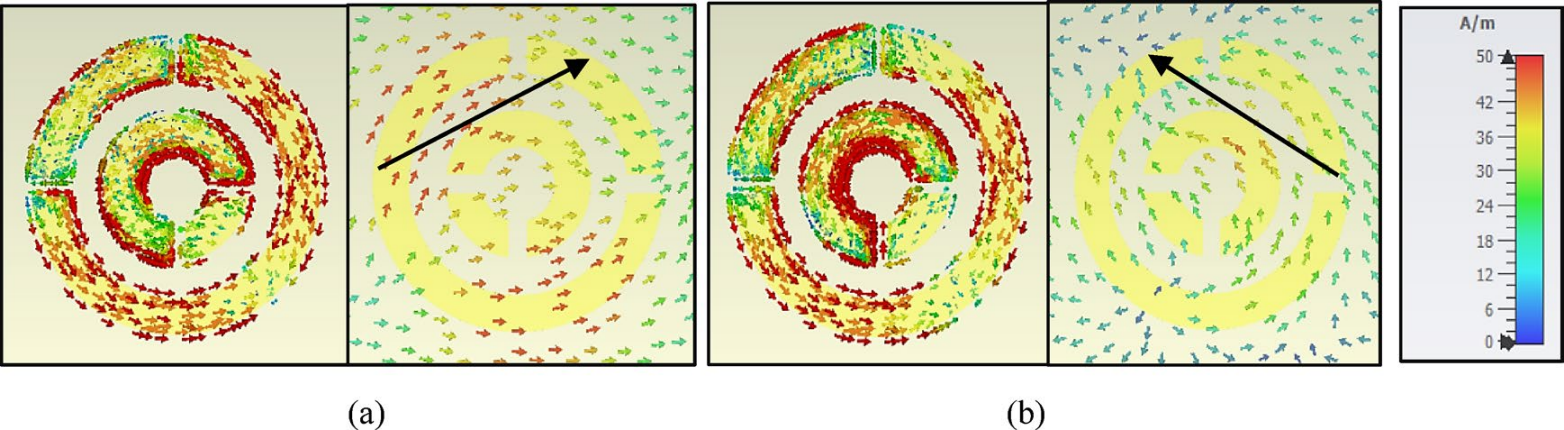
Surface current distributions on both the top and bottom planes at two unique resonance frequencies: (**a**) 20.84 GHz, (**b**) 24.58 GHz.

The physical mechanism of CPC is explored by examining surface current distribution on the MTS architecture and the bottom layer of a unit cell. Figure 10a, b show three plasmonic resonances (20.84 GHz and 24.58 GHz) on the highest (pattern layer) and back (ground) layers, respectively. The current is reduced intensively at the resonance frequency 20.84 GHz, as shown in Fig. 10a, but it is tightly distributed across the cell’s inner and outer circuits at the resonated frequencies 24.58 GHz, as shown in Fig. 10b.

#### Design validation

**Fig. 11:**
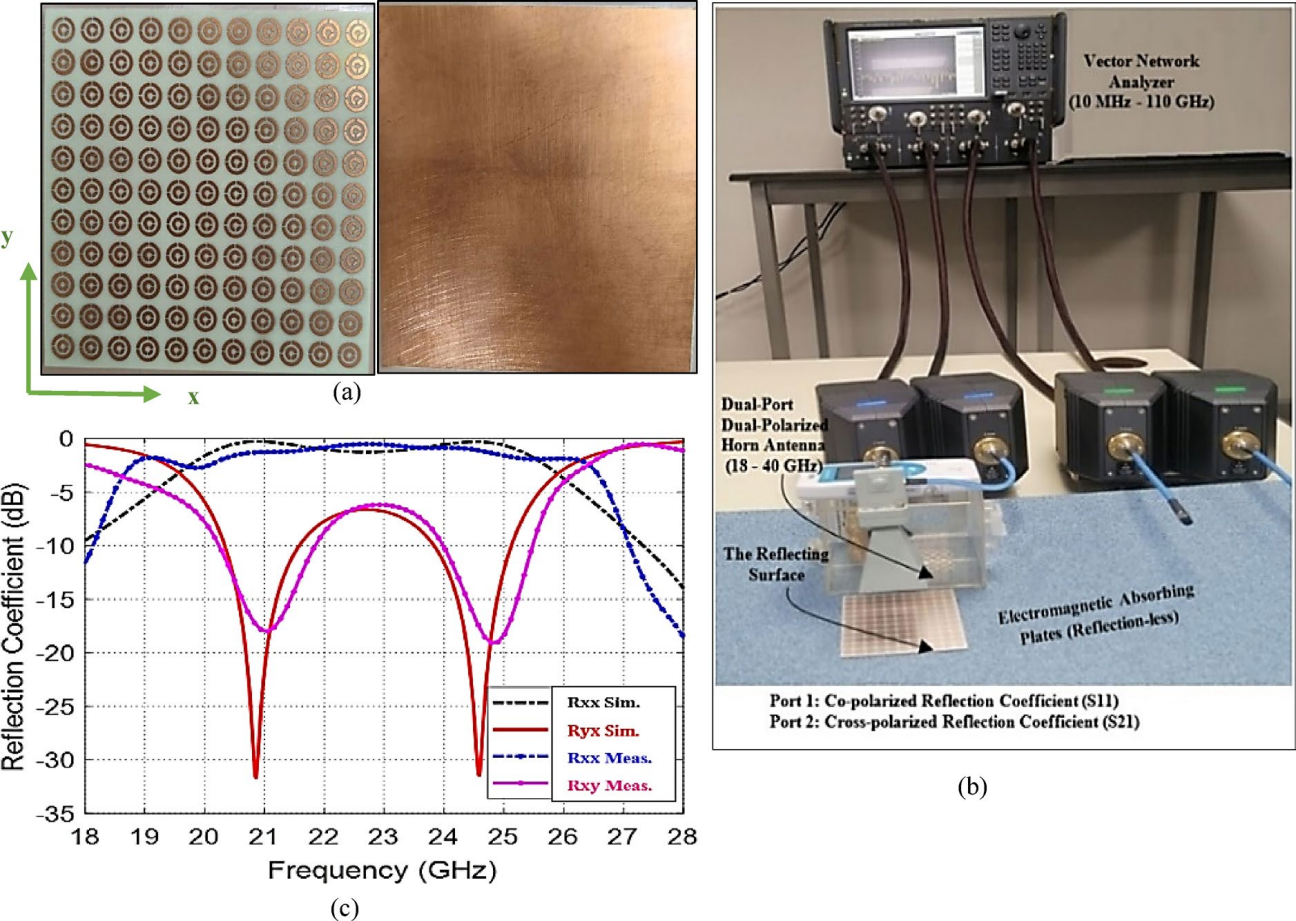
(**a**) The fabricated microwave CPC MTS, (**b**) Experimental setup for measuring the co-polarized and cross-polarized reflection coefficients of backscattering from the surface, (**c**) Variation of the co-polarized and cross-polarized reflection coefficients obtained by simulation and measurements.

The validate our simulation results by manufacturing an 11 × 11 unit-cell arrays on a copper-based Rogers 4350B substrate, dimensions 5.5 × 5.5 cm. The array size is sufficiently large that the periodic array approach is valid. Figure 11a depicts the completed design. The measuring setup employs wideband double-ridged antennas with horns (18–40 GHz) for both transmitting and receiving. The Agilent vector network analyzer (10 MHz–110 GHz) is used to measure scattering parameters within a anechoic chamber. Figure 11b depicts the whole measuring setup. The transverse polarization elements are measured by positioning the receiving horns horizontally or vertically. For example, to determine the co-polarization reflection coefficient ${R}_{yy}$, both antennas are vertically oriented along the *y*-axis. To measure the cross-polarization coefficient of reflection ${R}_{xy}$, the transmitting antenna is vertical, while the receiving antenna is horizontal (along the *x*-axis). ${R}_{xx}$ and ${R}_{yx}$ can be measured similarly. Figure 11c illustrates simulated and measured outcomes for co- and cross-polarized reflected when the transmitted wave is *x*-polarized at a bandwidth of (18–28 GHz); once more, experimental and simulated results accord well.

Table 2 compares the performance of the proposed design with other recently published related papers. It is found that the proposed design is very simple and provides much higher angular stability than some other cross-conversion systems. The designs^42,48^ have angular stability of 45° and 60° respectively, but the PCR is much lower than the proposed design. Although^33,41,51^ have high polarization efficiency, their angular stability is much lower than the proposed design.

**Table 2:** Comparison with other CPC MTS studies.

Designs	Applied design method	Dimensions (λ_0_)	Design complexity	Substrate with thickness	Operating bandwidth (GHz)	PCR	FBW (%)	Angular stability	Applications
				(electrical size)					
Ref^33^	MTM based defective ground structure (DGS)	0.36 × 0.36 × 0.14	Complex	FR4	5.4–22	90%	121%	Only for normal incidence wave	Radiometer, reflector antennas, remote sensors, and imaging sensors
				0.14 λ_o_					
Ref^41^	MTM polarization rotator (PR)	0.22 × 0.22 × 0.07	Complex	F4B-2	9.1–12.9	99%	34.5%	Only for normal incidence wave	Polarization manipulation and stealth technology
				0.07 λ_o_					
Ref^42^	MTM based SRR	0.18 × 0.18 × 0.06	Simple	FR4	5–10.8	60%	73%	Angularly stable up-to 45°	Amateur radio and satellite
				0.06 λ_o_					
Ref^48^	MTM based SRR	0.3 × 0.3 × 0.1	Simple	FR4	5–9.7 and 11.2–15	60%	63.9%	Angularly stable up-to 60°	Amateur radio and satellite
				0.1 λ_o_					
Ref^50^	MTM based triple-arrow resonant	0.22 × 0.22 × 0.11	Complex	FR4	8–18.5	90%	80%	Angularly stable up-to 45°	Radiometer, reflector antennas, remote sensors, and imaging sensors
				0.11 λ_o_					
Ref^51^	MTM based dipole shaped metal patch	0.45 × 0.45 × 0.1	Complex	FR4	7.1–8 and 13.3–25.8	~ 60–95%	63.9%	Only for normal incidence wave	Radiometer, reflector antennas, remote sensors, and imaging sensors
				0.1					
Present study	MTM based SRR	0.37 × 0.37 × 0.06	Simple	Rogers 4350B	19.6–25.9	90%	27.7%	Angularly stable up-to 45°	Stealth technology, EM measurements, and antenna construction
				0.06 λ_o_					

## Conclusion

A MTM technique is used to design absorber for solar infrared radiation. The design that is suggested comprises of two circular forms by rectangle gaps. The design was simulated and debated. The efficiency of the suggested meta-absorber design is evaluated, and simulation results show that it achieves nearly complete absorption at 13.29 THz, with absorption rates of 99%. It can also be easily adjusted to accommodate microwave frequency ranges, where a wide-band microwave CPC MTS is designed, simulated, fabricated, and tested. The proposed CPC unit cell structure depends on an artificial absorber design similar to a solar cell, but in the microwave region, The designed MTS converts *x*-polarized waves to *y*-polarized waves and inversely over a wide frequency range of (19.6–25.9 GHz). The polarization conversion efficiency is 100% at two plasmon resonances of 20.84 GHz and 24.58 GHz. The proposed MTS can be used in sensor applications, stealth technology, EM measurements, and antenna construction.

## Data Availability

The datasets used and/or analyzed during the current study available from the corresponding author on reasonable request.

## References

[1] Mulla, B. & Sabah, C. Perfect metamaterial absorber design for solar cell applications. *Waves Random Complex Media* **25**, 382–392 (2015).

[2] Bagmanci, M. et al. Solar energy harvesting with ultra-broadband metamaterial absorber. *Int. J. Mod. Phys. B* **33**, 1–16 (2019).

[3] Rufangura, P. & Sabah, C. Wide-band polarization independent perfect metamaterial absorber based on concentric rings topology for solar cellsapplication. *J. Alloys Compd.* **680**, 473–479 (2016).

[4] Hamdy, H. et al. Wavelength-selective metamaterial absorber based on 2D split rhombus grating for thermophotovoltic solar cell. *Opt. Quant. Electron.* **54**, 1–12 (2022).

[5] Dincer, F., Akgol, O., Karaaslan, M., Unal, E. & Sabah, C. Polarization angle independent perfect metamaterial absorbers for solar cell applications in the microwave, infrared, and visible regime. *Progress Electromagn. Res.* **144**, 93–101 (2014).

[6] Li, J. & Liu, Y. Mathematical simulation of metamaterial solar cells. *Adv. Appl. Math. Mech.* **3**, 702–715 (2011).

[7] John, V. D. H., Antony, B., Teja, N. N. & Gandikota, V*.* Wide band metamaterial absorber for gallium arsenide GaAs solar cells. In* International Conference on Signal Processing and Communication*, *ICPSC* 578–580 (2021).

[8] Alibakhshikenari, M. et al. A comprehensive survey on antennas on-chip based on metamaterial, metasurface, and substrate integrated waveguide principles for millimeter-waves and terahertz integrated circuits and systems. *IEEE Access* **10**, 3668–3692 (2022).

[9] Zhanga, Y. et al. Dual band visible metamaterial absorbers based on four identical ring patches. *Physica. E Low-Dimens. Syst. Nanostruct.* **127**, 114526 (2021).

[10] Yu, J., Lang, T. & Chen, H. All-metal terahertz metamaterial absorber and refractive index sensing performance. *Photonics* **8**, 1–8 (2021).

[11] Tao, H. et al. A dual band terahertz metamaterial absorber. *J. Phys. D Appl. Phys.* **43**, 1–5 (2010).

[12] Kerr, R. A. & Cho, A. Voilà! Cloak of invisibility unveiled. *AAAS* **314**, 403 (2006). 10.1126/science.314.5798.403 17053119

[13] Hamza, M. N. et al. Designing a high-sensitivity microscale triple-band biosensor based on terahertz MTMs to provide a perfect absorber for non-melanoma skin cancer diagnostic. *IEEE Photonics J.* **16**, 1–13 (2024).

[14] Vesalago, V. G. The electrodynamics of substances with simultaneously negative values of ∈ and μ. *Sov. Phys. Uspekhi* **10**, 509–514 (1968).

[15] Fang, N., Lee, H., Sun, C. & Zhang, X. Sub-diffraction-limited optical imaging with a silver superlens. *Science* **308**, 534–537 (2005). 15845849 10.1126/science.1108759

[16] Zhang, P. et al. Ultra-broadband tunable terahertz metamaterial absorber based on double-layer vanadium dioxide square ring arrays. *Artic. Micromach.* **13**, 1–12 (2022). 10.3390/mi13050669 PMC9145387 35630136

[17] Ustunsoy, M. P. & Sabah, C. Dual-band high-frequency metamaterial absorber based on patch resonator for solar cell applications and its enhancement with graphene layers. *J. Alloys Compd.* **143**, 1–19 (2016).

[18] Tang, J., Xiao, Z. & Xu, K. Ultra-thin metamaterial absorber with extremely bandwidth for solar cell and sensing applications in visible region. *J. Elsevier* **60**, 142–147 (2016).

[19] Fang, S. et al. Design and experimental realization of triple-band filtering metamaterial in sub-terahertz band enabled by conductivity coupling response of two identical split rings. *J. Elsevier Sci. Direct* **183**, 112345 (2025).

[20] Wang, X. B. et al. Design and experimental realization of triple-band electromagnetically induced transparency terahertz metamaterials employing two big-bright modes for sensing—applications. *J. Nanoscale* **45**, 18435–18446 (2023). 10.1039/d3nr05095e 37937951

[21] Montaser, A. M. Design of multiband PIFA with low SAR value for all commercial mobile communication bands. *Int. J. RF Microw. Comput. Aided Eng.* **25**, 194–201 (2015).

[22] Sabaawi, A. M. A., Tsimenidis, C. C. & Sharif, B. S. Infra-red nano-antennas for solar energy collection. In 2011 *Loughborough Antennas & Propagation Conference *1–4 (IEEE, 2011).

[23] Bozzetti, G. et al. Analysis and design of a solar rectenna. In 2010 *J. IEEE International Symposium on Industrial Electronics (ISIE)* 4–7 (IEEE, 2010).

[24] Berland, B. Photovoltaic technologies beyond the horizon: Optical rectenna solar cell. *Natl Renew. Energy Laborator (NREL)* 1–24 (2003).

[25] Z. El Dein, A., Abdel-Rahman, A. B., Fat-Helbary, R. E. & Montaser, A. M. Tunable—compact band stop defected ground structure (DGS) with lumped element. In *7th International Conference of Multi-Conference on systems, signals and Device* (2010).

[26] Montaser, A. M., Abdel-Rahman, A. B., Elmikati, H. A. & Mahmoud, K. R. Design bluetooth and notched-UWB e-shape antenna using optimization techniques. *Prog. Electromagn. Res. B PIER* **47**, 279–295 (2013).

[27] Mahmoud, K. R., Baz, A., Alhakami, W., Alhakami, H. & Montaser, A. M. The performance of circularly polarized phased sub-array antennas for 5g laptop devices investigating the radiation effects. *Progress Electromagn. Res. (PIER) C* **110**, 267–283 (2021).

[28] Ghattas, A. S. W., Saad, A. A. R. & Khaled, E. E. M. Compact patch antenna array for 60 GHz millimeter-wave broadband applications. *Wirel. Pers. Commun.* **114**, 2821–2839 (2020).

[29] Rufangura, P. & Sabah, C. Design and characterization of a dual-band perfect metamaterial absorber for solar cell applications. *J. Alloy. Compd.* **671**, 43–50 (2016).

[30] Akbari, M., Farahani, M., Sebak, A. R. & Denidni, T. A. Ka-band linear to circular polarization converter based on multilayer slab with broadband performance. *IEEE Access* **5**, 17927–17937 (2017).

[31] Akbari, M., Abo Ghalyon, H., Farahani, M., Sebak, A. R. & Denidni, T. Spatially decoupling of CP antennas based on FSS for 30-ghz mimo systems. *IEEE Access* **5**, 6527–6537 (2017).

[32] Akbari, M. S., Farahani, M., Sebak, A. R. & Denidni, T. Gain enhancement of circularly polarized dielectric resonator antenna based on FSS superstrate for MMW applications. *IEEE Trans. Antennas Propag.* **16**, 2324–2327 (2017).

[33] Moghadam, M. J., Akbari, M., Samadi, F. & Sebak, A. R. Wideband cross polarization rotation based on reflective anisotropic surfaces. *IEEE Access* **6**, 15919–15925 (2018).

[34] Yu, N. et al. Flat Optics: Controlling wavefronts with optical antenna metasurfaces. *IEEE J. Sel. Top. Quantum Electron.* **19**, 4700423 (2013).

[35] Grady, N. K. et al. Terahertz metamaterials for linear polarization conversion and anomalous refraction. *Science* **340**, 1304–1307 (2013). 23686344 10.1126/science.1235399

[36] Li, G. et al. Spin-enabled plasmonic metasurfaces for manipulating orbital angular momentum of light. *Nano. Lett.* **13**, 4148–4151 (2013). 23965168 10.1021/nl401734r

[37] Yang, Y. et al. Dielectric meta-reflectarray for broadband linear polarization conversion and optical vortex generation. *Nano. Lett.* **14**, 1394–1399 (2014). 24547692 10.1021/nl4044482

[38] Hong-Yu, C. et al. Broadband perfect polarization conversion metasurfaces. *Chin. Phys. B* **24**, 014201 (2015).

[39] Sun, W. et al. A transparent metamaterial to manipulate electromagnetic wave polarizations. *Opt. Lett.* **36**, 927–929 (2011). 21403731 10.1364/OL.36.000927

[40] Ma, H. F. et al. Broadband circular and linear polarization conversions realized by thin birefringent reflective metasurfaces. *Opt. Mater. Express* **4**, 1717–1724 (2014).

[41] Zheng, Q., Guo, C., Li, H. & Ding, J. Wideband and high efficiency reflective polarization rotator based on metasurface. *J. Electromagn. Waves Appl.* **32**, 265–273 (2017).

[42] Ismail Khan, M., Fraz, Q. & Tahir, F. A. Ultra-wideband cross polarization conversion metasurface insensitive to incidence angle. *J. Appl. Phys.* **121**, 1–9 (2017).

[43] Zhao, Y. & Al`u, A. Tailoring the dispersion of plasmonic nanorods to realize broadband optical meta-waveplates. *Nano. Let.* **13**, 1086–1091 (2013). 23384327 10.1021/nl304392b

[44] Sieber, P. E. & Werner, D. H. Reconfigurable broadband infrared circularly polarizing reflectors based on phase changing birefringent metasurfaces. *Opt. Express* **21**, 1087–1100 (2013). 23389002 10.1364/OE.21.001087

[45] Wu, L. et al. Dualband asymmetric electro-magnetic wave transmission for dual polarizations in chiral metamaterial structure. *Appl. Phys. B* **117**, 527–531 (2014).

[46] Zhu, H. L., Cheung, S. W., Chung, K. L. & Yuk, T. I. Linearto-circular polarization con- version using metasurface. *IEEE Trans. Antennas Propag.* **61**, 4615–4623 (2013).

[47] Kundu, D., Mohan, A. & Chakrabarty, A. Ultrathin high-efficiency X-band reflective polarization converter using sunken double arrowhead metasurface. *Asia-Pacific Microwave Conference (APMC)* 1–4 (*IEEE*, 2016).

[48] Ismail Khan, M. & Tahir, F. A. An angularly stable dual-band anisotropic cross polarization conversion metasurface. *Appl. Phys.* **122**, 1–9 (2017).

[49] Pandey, A. & Rana, S. B. Review of metamaterials, types and design approaches. *Inter. J. Eng. Sci.* **17**, 359–363 (2016).

[50] Rashid, A., Murtaza, M., Zaidi, S., Zaki, H. & Tahir, F. A single-layer, wideband and angularly stable metasurface based polarization converter for linear-to-linear cross-polarization conversion. *PLOS ONE* 1–11 (2023). 10.1371/journal.pone.0280469 PMC9858438 36662794

[51] Khan, B. et al. Design and experimental analysis of dual-band polarization converting metasurface for microwave applications. *Sci. Rep.* **10**, 1–14 (2020). 32958835 10.1038/s41598-020-71959-y PMC7505988

[52] Ismail Khan, M. & Tahir, F. A. A broadband cross-polarization conversion anisotropic metasurface based on multiple plasmon resonances. *Chin. Phys. B* **27**, 1–9 (2018).

